# Comparison of Trends in Rates of Sexually Transmitted Infections Before vs After Initiation of HIV Preexposure Prophylaxis Among Men Who Have Sex With Men

**DOI:** 10.1001/jamanetworkopen.2020.30806

**Published:** 2020-12-23

**Authors:** Hamish McManus, Andrew E. Grulich, Janaki Amin, Christine Selvey, Tobias Vickers, Benjamin Bavinton, Iryna Zablotska, Stephanie Vaccher, Fengyi Jin, Joanne Holden, Karen Price, Barbara Yeung, Gesalit Cabrera Quichua, Erin Ogilvie, Anna McNulty, David Smith, Rebecca Guy

**Affiliations:** 1 Kirby Institute, University of New South Wales Sydney, Sydney, New South Wales, Australia; 2 Department of Health Systems and Populations, Macquarie University, Sydney, New South Wales, Australia; 3 Ministry of Health, New South Wales Government, St Leonards, New South Wales, Australia; 4 Westmead Clinical School, Sydney University, Sydney, New South Wales, Australia; 5 AIDS Council New South Wales, Sydney, New South Wales, Australia; 6 Sydney Sexual Health Centre, Sydney, New South Wales, Australia; 7 North Coast HIV/Sexual Health Services, Lismore, New South Wales, Australia

## Abstract

**Importance:**

There have been concerns that HIV preexposure prophylaxis (PrEP) may be associated with increases in sexually transmitted infections (STIs) because of subsequent reductions in condom use and/or increases in sexual partners.

**Objective:**

To determine trends in STI test positivity among high-risk men who have sex with men (MSM) before and after the start of HIV PrEP.

**Design, Setting, and Participants:**

A before-after analysis was conducted using a subcohort of a single-group PrEP implementation study cohort in New South Wales, Australia (Expanded PreEP Implementation in Communities in New South Wales [EPIC-NSW]), from up to 1 year before enrollment if after January 1, 2015, and up to 2 years after enrollment and before December 31, 2018. STI testing data were extracted from a network of 54 sexual health clinics and 6 primary health care clinics Australia-wide, using software to deidentify, encrypt, and anonymously link participants between clinics. A cohort of MSM dispensed PrEP for the first time during the study, with 2 or more STI tests in the prior year and who tested during follow-up, were included from the EPIC-NSW cohort of HIV-negative participants with high-risk sexual behavior. Data analysis was performed from June to December 2019.

**Exposures:**

Participants were dispensed coformulated tenofovir disoproxil fumarate (300 mg) and emtricitabine (200 mg) as HIV PrEP.

**Main Outcomes and Measures:**

The main outcome was STI, measured using test positivity, defined as the proportion of participants testing positive for an STI at least once per quarter of follow-up. Outcomes were calculated for *Chlamydia trachomatis* and *Neisseria gonorrhoea* by site of infection (anorectal, pharyngeal, urethral, or any) and for syphilis.

**Results:**

Of the EPIC-NSW cohort of 9709 MSM, 2404 were included in the before-after analysis. The mean (SD) age of the participants was 36 (10.4) years, and 1192 (50%) were Australia-born. STI positivity was 52% in the year after PrEP (23.3% per quarter; 95% CI, 22.5%-24.2% per quarter) with no significant trend (mean rate ratio [RR] increase of 1.01 per quarter [95% CI, 0.99-1.02]; *P* = .29), compared with 50% positivity in the year prior to PrEP (20.0% per quarter [95% CI, 19.04%-20.95% per quarter]; RR for overall STI positivity, 1.17 [95% CI, 1.10-1.24]; *P* < .001), with an increase in quarterly STI positivity (mean RR of 1.08 per quarter, or an 8% increase per quarter [95% CI, 1.05-1.11]; *P* < .001; RR, 0.93 [95% CI, 0.90-0.96]; *P* < .001). Findings were similar when stratified by specific STIs and anatomical site.

**Conclusions and Relevance:**

STI rates were high but stable among high-risk MSM while taking PrEP, compared with a high but increasing trend in STI positivity before commencing PrEP. These findings suggest the importance of considering trends in STIs when describing how PrEP use may be associated with STI incidence.

## Key Points

**Question:**

Is access to HIV preexposure prophylaxis (PrEP) associated with the trajectory of preexisting trends in sexually transmitted infections (STIs) among high-risk men who have sex with men (MSM)?

**Findings:**

In a before-after cohort study of high-risk MSM participating in a population-based PrEP implementation project, STI positivity was high while men were taking PrEP with no increasing trend in quarterly STI positivity, compared with similar high positivity before PrEP but an increase of 8% per quarter.

**Meaning:**

These findings suggest that studies that do not consider preexisting STI trends may overestimate the association of PrEP with STI incidence.

## Teaser

This cohort study examines trends in sexually transmitted infection (STI) test positivity among high-risk men who have sex with men before and after the start of preexposure prophylaxis (PrEP) for HIV.

## Introduction

HIV preexposure prophylaxis (PrEP) is a key prevention approach to eliminating HIV transmission. Use of PrEP is increasing in men who have sex with men (MSM) and other populations at high risk of HIV infection.^1^ In some parts of the world, the benefits of expanding access to PrEP for MSM (combined with treatment as prevention) are being realized, with a 32% reduction in recent HIV infection in MSM at the state-level in New South Wales, Australia, after rapid rollout over a 12-month period.^2^ However, there have been concerns that use of PrEP may be followed by increased incidence of other sexually transmitted infections (STIs), such as chlamydia, gonorrhea, and infectious syphilis, because of a reduction in condom use and/or increase in sexual partners.^3^ In several high-income countries, PrEP rollout has been temporally associated with an increase in reporting of STI notifications among MSM.^4,5,6^

A 2018 meta-analysis^6^ that included 4388 MSM enrolled in 8 open-label PrEP studies compared STI incidence before and after PrEP initiation and reported a nonsignificant increase in STI diagnoses overall (odds ratio, 1.24 [95% CI, 0.99-1.54]). A 2019 meta-analysis^7^ of epidemiological characteristics of STIs in PrEP users found high pooled prevalence of STI at initiation (23.9% [95% CI, 18.6%-29.6%]) and high pooled incidence during PrEP (72.2 cases per 100 person-years [95% CI, 60.5-86.2 cases per 100 person-years]). In Australia, the US, and the United Kingdom, increasing trends in notifications of STI diagnoses were observed over several years before PrEP being broadly available, including in MSM populations.^8,9,10,11,12,13^ Furthermore, increased frequency of STI testing, particularly that associated with PrEP, may also lead to an increase in STI diagnoses even if incidence is not increasing.^14^

In New South Wales, Australia, between 2016 and 2018, a large-scale PrEP access program (Expanded PreEP Implementation in Communities in New South Wales [EPIC-NSW]) rapidly rolled out PrEP to almost 10 000 MSM at high risk of HIV infection. This network provided a unique opportunity to determine whether access to PrEP is associated with changes in the trajectory of the preexisting trend in STIs among MSM in New South Wales. We examined the magnitude and direction of trends in STIs in a cohort of high-risk MSM men taking PrEP and compared trends during the PrEP period with trends in the same men before PrEP scale-up.

## Methods

### EPIC-NSW

The methods of the EPIC-NSW implementation study have been published elsewhere.^15^ EPIC-NSW was a single-group, open-label, cohort study on the use of once-daily, coformulated tenofovir disoproxil fumarate (300 mg) and emtricitabine (200 mg) as HIV-PrEP in individuals at high risk of HIV infection. Written informed consent was obtained from all participants. The study was approved by the ethics committee of St Vincent’s Hospital. This study follows the Strengthening the Reporting of Observational Studies in Epidemiology (STROBE) reporting guideline.^16^

EPIC-NSW inclusion criteria were based on participants reporting ongoing risk and at least 1 of the following behavioral criteria (relating to the last 3 months): (1) receptive condomless anal intercourse with casual male partners of HIV-positive or unknown status; (2) diagnosis of rectal chlamydia, rectal gonorrhea, or infectious syphilis; (3) use of crystal methamphetamine; or (4) condomless anal intercourse with an HIV-positive regular partner who did not have an undetectable HIV viral load. As per Australian PrEP guidelines,^17^ men were required to have a negative HIV test at baseline. HIV testing was recommended 1 month after initiation and every 3 months thereafter. Comprehensive STI testing, consisting of nucleic acid–based testing for urethral, anorectal, and pharyngeal gonorrhea and chlamydia and serological testing for syphilis, was recommended at baseline and at every 3 months.

### Study Design, Setting, and Population

We performed a before-after analysis of participants in New South Wales, Australia, enrolled across the 30 EPIC-NSW clinics between March 1, 2016, and April 30, 2018. Inclusion was based on participants who had data available for 2 or more STI tests in the year before EPIC-NSW enrollment and at least 1 STI test following enrollment, to minimize any impact of increased STI testing on STI detection before and after the start of PrEP use. Inclusion in this study was limited to MSM because the majority (99%) of EPIC-NSW participants are male and only 0.5% report heterosexual identity.^18^ Participants who reported PrEP use in the 3 months before enrollment, through personal importation, were excluded from the analysis, to ensure that the data in the year before the enrollment window reflected trends in men who were not taking PrEP. Baseline was set as the 90-day interval of 45 days before and after the date of enrollment. Participants were followed from the later of January 1, 2015, or 12 months before the baseline interval, until up to 24 months after baseline, with follow-up censored on December 31, 2018.

### Data Sources

STI testing data were extracted from the larger Australian Collaboration for Coordinated Enhanced Sentinel Surveillance of Sexually Transmissible Infections (ACCESS) network (54 sexual health clinics and 6 primary health care clinics Australia-wide) of which EPIC enrollment clinics are a subset, using GRHANITE client software version 1.5.6521 (The University of Melbourne) to deidentify, encrypt, and anonymously link participants between all clinics in the ACCESS network.^19,20^ In 4 non-ACCESS clinics, data were entered manually into a study database and were not able to be linked with other clinic data. Syphilis diagnosis data were extracted for a subset of participants attending publicly funded clinics where clinical and laboratory information were recorded systematically in the electronic patient medical records.

### Study Outcomes

The study outcome was STI measured using STI test positivity. STI test positivity for each STI test was defined as the proportion of participants who tested positive for that test at least once in a quarterly window of follow-up. Outcomes were calculated for each organism tested for: *Chlamydia trachomatis* and *Neisseria gonorrhoea* by site of infection (anorectal, pharyngeal, urethral, or any), and for the clinical diagnoses of infectious syphilis, determined by the clinics using a combination of current and past serological and clinical information. Despite infectious syphilis being a diagnosis rate, we used the term *positive* throughout. Positivity was used for the primary outcome, because, although it reflects incidence trends, calculating positivity does not require an initial negative test result for entry into analyses and does not assume a negative status of untested individuals.

### Statistical Analysis

#### Descriptive Analysis

Participant testing characteristics were tabulated, including age (mean [SD]), age group (18-24, 25-34, 35-44, and ≥45 years), sexual identity (gay or bisexual), country of birth (Australia; Canada, Ireland, New Zealand, US, or UK; Asia; Europe excluding UK and Ireland; Africa; South America, Central America, or Caribbean; or other), prevalence of gay men in the postal code where the participant resides (≥20%, 10% to <20%, 5% to <10%, <5%, and missing), and type of recruiting site (private sexual health clinic, public clinic, or hospital). Descriptive testing characteristics were evaluated, including testing frequency, testing intervals, and repeat positivity. Differences between groups were assessed using *t* tests for comparison of means, and χ^2^ tests for comparison of categorical data. All tests were 2-sided. Significance was set at *P* < .05.

#### Trends Analysis

Multivariable random effects log-binomial regression models were used to assess quarterly trends in STI positivity for the year before PrEP commencement inclusive of the enrollment window compared with the trend in the 2 years after enrollment, as well as any discontinuity in trend associated with a concurrent increase or decrease (upward or downward location shift) in STI positivity associated with PrEP status. We used a binary time-updated indicator of enrollment status and an interaction between this term and a continuous time covariate (quarter from enrollment), with participant included as random effect. This enabled us to test for potential change in positivity attributable to immediate change in sexual practices (location shift) after adjusting for marginal change in time trend following PrEP, a pattern unable to be detected by a time trend alone. Models were also fit without location shift adjustment for comparison with full models. Two sensitivity analyses were conducted: first, we excluded the enrollment window to reduce possible effects of increased case finding and change in risk behavior associated with the EPIC-NSW screening and enrollment period; and second, we adjusted for time period of enrollment (early [before July 2016], middle [July 2016 to December 2016], and late [January 2017 to April 2018]).

To allow comparison of results with models that do not adjust for trend, we assessed the difference in STI positivity fit without a trend (mean positivity) before and after EPIC-NSW enrollment using Poisson regression models. Poisson regression was used instead of binomial regression because, although participants were restricted to a maximum of 1 positive case per quarter as per the primary analysis, participants may have had more than 1 quarter with positive results. Data analysis was performed using Stata statistical software version 15.1 (StataCorp) from June to December 2019.

## Results

**Figure 1.**
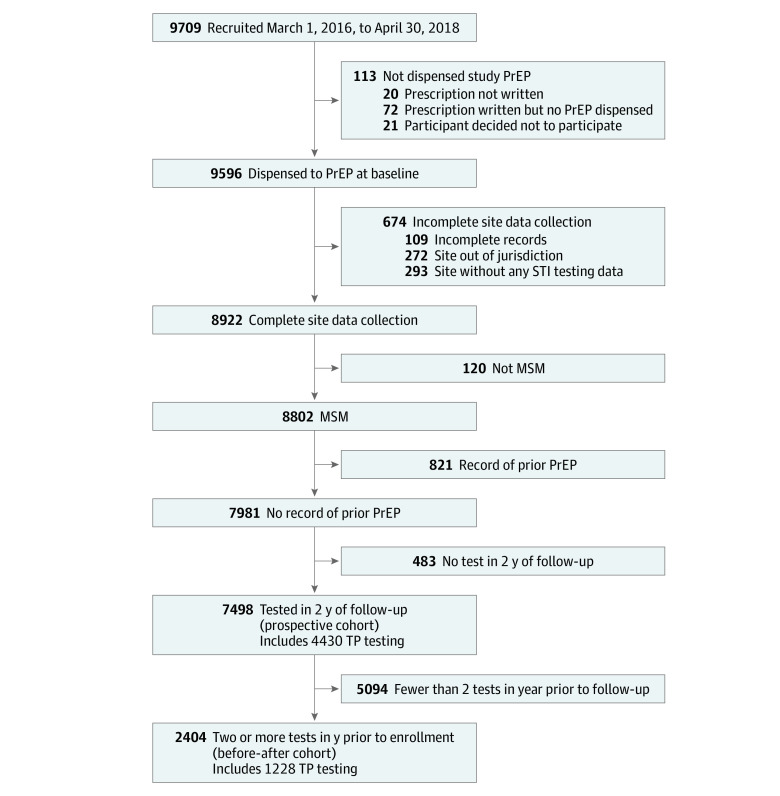
Study Enrollment Flowchart MSM indicates men who have sex with men; PrEP, preexposure prophylaxis; STI, sexually transmitted infection; and TP, *Treponema pallidum*.

A total of 9709 participants were recruited, of whom 9596 (99%) were dispensed PrEP. Of these, 8922 (93%) had complete data available, with 8802 (99%) retained on the basis of identified MSM status and a cohort of 7981 (90.7%) not having recorded prior PrEP use. Among 7498 of these men (94%) who had recorded testing in the 2 years of follow-up, 4430 (59%) had recorded syphilis testing. The before-after cohort was composed of 2404 men (32%) with 2 or more tests in the year before enrollment, of whom 1228 (51%) had recorded infectious syphilis testing (Figure 1).

### Participant Characteristics

The mean (SD) age of the cohort was 36 (10.4) years, with 1017 men (42%) in the 25 to 34 years age group and 2260 (94%) identifying as gay or homosexual. One-half (1192 men [50%]) were Australia-born, and most (1403 men [59%]) lived in postal codes with more than 5% of the population gay men. One-half of the cohort (1214 men [50%]) were enrolled at public sexual health clinics, and 1393 (58%) were enrolled at least 2 years before the cohort censoring date (Table 1).

**Table 1.** Characteristics of Before-After Cohort Participants at Enrollment

Characteristic	Participants, No. (%) (N = 2404)^a^
Age, mean (SD), y	35.6 (10.4)
Age group, y	
18-24	285 (12)
25-34	1017 (42)
35-44	639 (27)
≥45	463 (19)
Sexual identity	
Bisexual	144 (6)
Gay or homosexual	2260 (94)
Country of birth	
Australia	1192 (50)
Canada, Ireland, New Zealand, US, or UK	287 (12)
Asia	350 (15)
Europe (excluding UK and Ireland)	103 (4)
Africa	31 (1)
South America, Central America, or Caribbean	100 (4)
Other	55 (2)
Missing	286 (12)
Gay men in postal code, %	
≥20	831 (35)
10 to <20	268 (11)
5 to <10	304 (13)
<5	1000 (42)
Missing	1 (0)
Type of recruiting site	
Public sexual health clinic	1214 (50)
Private	1085 (45)
Hospital	105 (4)
Enrollment period	
May to June 2016 (early)	724 (30)
July to December 2016 (middle)	669 (28)
January to 2017 to April 2018 (late)	1011 (42)

^a^ Includes participants with testing after enrollment, no record of prior preexposure prophylaxis, and 2 or more tests in the year before enrollment; 587 of 30 549 participants (1.9%) did not have specified anatomical site.

### STI Testing

STI testing frequency increased from a mean (SD) of 3.22 (1.44) tests per year in the year prior to PrEP use to 3.57 (1.79) tests per year in the 2 years after EPIC-NSW enrollment (difference, 0.35 test per year; 95% CI, 0.26 to 0.44 test per year; *P* < .001), with higher frequency in the first year after PrEP use (mean [SD], 4.45 [1.85]; difference, 1.23 tests per year; 95% CI, 1.14 to 1.32 tests per year; *P* < .001) (eTable 1 in the Supplement). The proportion of participants with a positive STI result was 50% in the year prior to PrEP (20.0% per quarter; 95% CI, 19.04% to 20.95% per quarter) and 52% (23.3% per quarter; 95% CI, 22.5% to 24.2% per quarter) in the year after PrEP (difference, 1.4%; 95% CI, −1% to 4%; *P* = .34), and 63% had a positive STI result during 2 years of follow-up. The proportion of participants with 2 or more positive results was 16% before PrEP compared with 20% over a comparable duration (1 year) after PrEP.

### Trend Analysis of Quarterly STI Positivity

**Figure 2.**
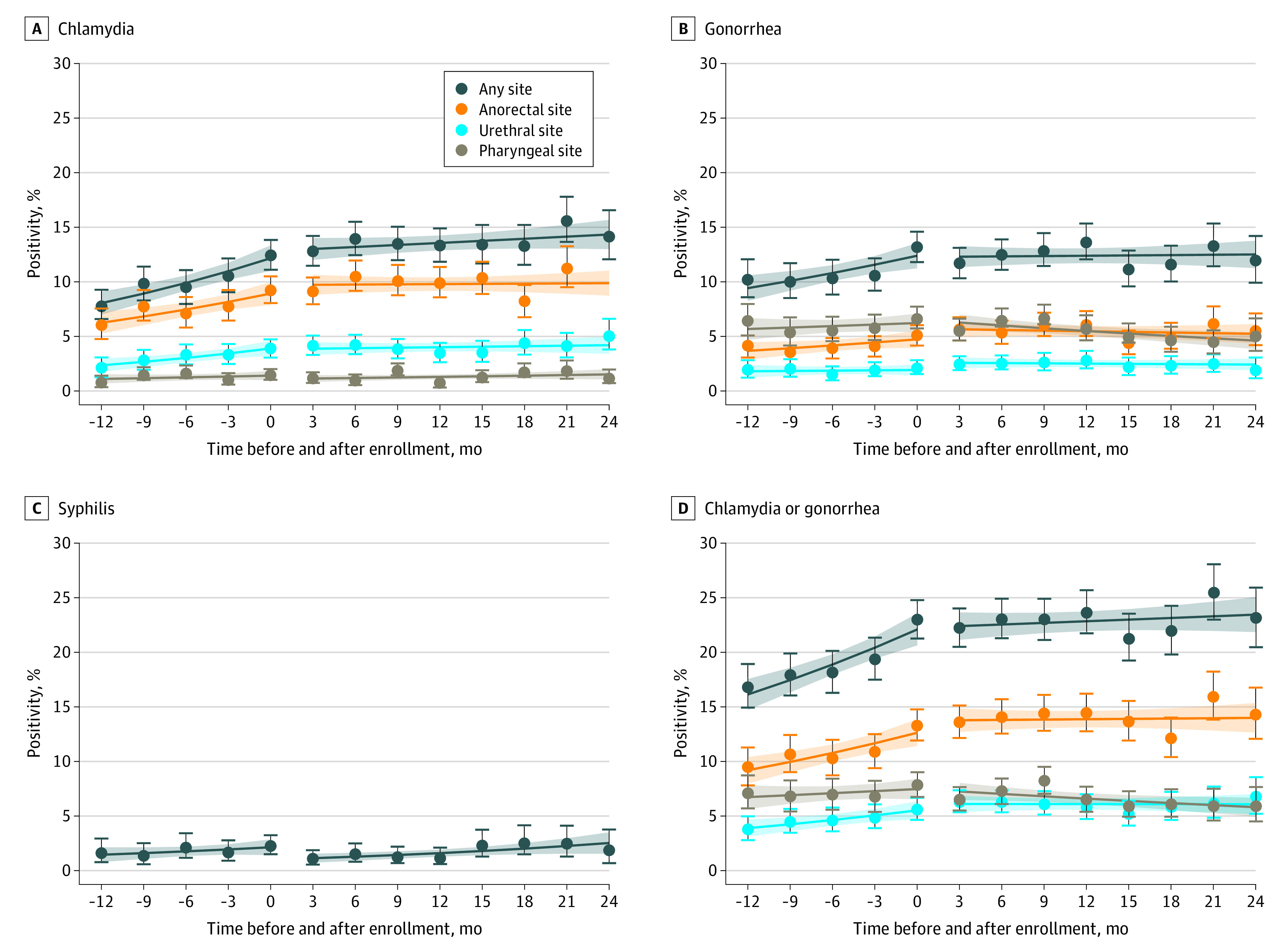
Sexually Transmitted Infection Positivity Before and After Commencement of Preexposure Prophylaxis (PrEP) in 2404 Patients With No Prior PrEP Use and With 2 or More Tests in Year Prior to PrEP Circles with error bars represent observed quarterly positivity and 95% CIs. Solid lines represent trend, and shaded areas represent 95% CIs for trend.

Mean *C trachomatis* or *N gonorrhoea* positivity was 19.5% per quarter (95% CI, 18.56%-20.46% per quarter) before PrEP including baseline, and 22.9% per quarter (95% CI, 22.06%-23.71% per quarter) after PrEP, representing a 17% increase (rate ratio [RR], 1.17; 95% CI, 1.10-1.24; *P* < .001) (Figure 2 and eTable 2 in the Supplement). In the period before PrEP enrollment, there was an increasing trend in quarterly *C trachomatis* or *N gonorrhoea* positivity (RR increase, 1.08 per quarter [or an 8% increase per quarter]; 95% CI, 1.05-1.11 per quarter; *P* < .001); however, no trend was observed after starting PrEP (RR increase, 1.01 per quarter; 95% CI, 0.99-1.02 per quarter; *P* = .29). The rate of increase in combined *C trachomatis *or *N gonorrhoea* positivity after starting PrEP was less than the rate of increase before starting PrEP (RR, 0.93; 95% CI, 0.90-0.96; *P* < .001) (Table 2). There was no difference in positivity trends before and after PrEP for infectious syphilis (RR, 1.02; 95% CI, 0.85-1.22; *P* = .85). No upward or downward change in positivity (location shift) for *C trachomatis* or *N gonorrhoea* at the time of enrollment was detected when trend adjustment was made (RR, 1.01; 95% CI, 0.92-1.10; *P* = .88) (eTable 3 in the Supplement). Models of trend fit without location shift adjustment were qualitatively similar to adjusted models (eTable 4 in the Supplement).

**Table 2.** Before-After Trend in Positivity for Participants With No Record of Prior PrEP Use and 2 or More Tests in Year Before PrEP^a^

Disease and site of infection	Before PrEP, change/quarter (95% CI), %	*P* value^b^	After PrEP, change/quarter (95% CI), %	*P* value^b^	RR (95% CI)	*P* value^b^
Chlamydia						
Anorectal	1.09 (1.03-1.15)	.002	1.00 (0.98-1.03)	.86	0.92 (0.86-0.97)	.005
Pharyngeal	1.06 (0.93-1.22)	.38	1.04 (0.97-1.12)	.25	0.98 (0.84-1.14)	.80
Urethral	1.13 (1.04-1.23)	.004	1.01 (0.97-1.05)	.57	0.89 (0.81-0.98)	.02
Any site	1.11 (1.06-1.16)	<.001	1.01 (0.99-1.03)	.16	0.91 (0.87-0.96)	<.001
Gonorrhea						
Anorectal	1.07 (0.99-1.15)	.11	0.99 (0.96-1.02)	.53	0.93 (0.85-1.01)	.09
Pharyngeal	1.02 (0.96-1.09)	.47	0.96 (0.93-0.99)	.01	0.94 (0.87-1.004)	.07
Urethral	1.02 (0.91-1.13)	.78	0.99 (0.95-1.04)	.70	0.98 (0.87-1.10)	.68
Any site	1.07 (1.02-1.12)	.002	1.00 (0.98-1.02)	.81	0.94 (0.89-0.98)	.007
Chlamydia or gonorrhea						
Anorectal	1.08 (1.03-1.13)	.001	1.00 (0.98-1.02)	.82	0.93 (0.88-0.97)	.002
Pharyngeal	1.03 (0.97-1.09)	.36	0.97 (0.94-1.00)	.04	0.94 (0.89-1.01)	.08
Urethral	1.09 (1.02-1.17)	.01	1.00 (0.97-1.03)	.97	0.92 (0.85-0.99)	.02
Any site	1.08 (1.05-1.12)	<.001	1.01 (0.99-1.02)	.35	0.93 (0.90-0.96)	<.001
Syphilis	1.10 (0.94-1.29)	.24	1.12 (1.02-1.23)	.01	1.02 (0.85-1.22)	.85
Any disease, any site	1.08 (1.05-1.11)	<.001	1.01 (0.99-1.02)	.29	0.93 (0.90-0.96)	<.001

Abbreviations: PrEP, Preexposure prophylaxis; RR, rate ratio.

^a^ A total of 2404 EPIC-NSW participants with testing after enrollment and no record of prior PrEP and with 2 or more test in the year prior to enrollment were included; 587 of 30 549 tests (1.9%) did not have specified anatomical site, including 263 of 12 733 (2.1%) before PrEP and 324 of 17 492 (1.8%) during PrEP (χ^2^ test for difference, *P* = .12). Multivariable binomial-log regression was adjusted for PrEP status and quarter of follow-up, which are patient-level random effects. Intervention effect RRs are available in eTable 3 in the Supplement.

^b^ Wald test.

### Sensitivity Analyses of STI Positivity

A sensitivity analysis excluding STIs diagnosed in the 90-day enrollment window found no change in trend for *C trachomatis* or *N gonorrhoea* positivity (RR, 0.97; 95% CI, 0.92-1.01; *P* = .16), including by type of infection and STI-specific site of infection (eTable 5 in the Supplement), and was qualitatively similar to primary analyses except for an detected increase (location shift) in *N gonorrhoea* anorectal positivity (RR, 1.46; 95% CI, 1.04-2.05; *P* = .03). A sensitivity analysis adjusting for period of enrollment found no association between enrollment period and positivity and was qualitatively similar to primary analyses (eTable 6 in the Supplement).

## Discussion

STI positivity was very high during scale-up of PrEP in a high-risk population of HIV-negative MSM in New South Wales participating in an implementation trial. In our analysis of men who had retrospective data on STI testing before PrEP, the proportion with a positive STI result was 50% in the year before PrEP, with a mean quarterly increase in STI positivity of 8%. After commencing PrEP, STI positivity rates remained high with a cumulative STI positivity of 52%, but there was no significant change in quarterly STI positivity. This finding suggests that previously described increasing incidence of STIs among men taking PrEP mainly reflects a preexisting increasing trend in STIs in these men. These findings suggest that analyses that do not adjust for preexisting trends in STIs may incorrectly associate PrEP use with increasing STI incidence.

Our findings indicate that men targeted for PrEP were at high and increasing risk of STIs before initiating PrEP. Although the reasons for this are not well established, they have been correlated with increases in condomless anal intercourse and numbers of sexual partners^6,8,21^ and associated with HIV biomedical strategies, such as treatment used as prevention.^22,23^ The main difference between our studies and others is that we found that the STI test positivity rate after commencing PrEP stabilized. These findings are consistent with those of an international meta-analysis^24^ of PrEP use that found no change in the proportion of MSM reporting condomless sex from baseline to follow-up while taking PrEP, which suggests that groups of study participants use condoms inconsistently and regardless of PrEP use. The reason for the difference is that other studies have generally focused on concurrent differences in STI rates in men taking PrEP vs other men not taking PrEP.^25^ More recent reviews^7^^,^^24^ have focused on differences in STI rates in men at baseline vs STI rates when taking PrEP. However, those studies do not include a control period to establish prior trend, and, therefore, PrEP cannot not be attributed to observed increases from baseline.

Increases in testing frequency in this study were observed after enrollment, as most men were tested for STIs every 3 months while taking PrEP, and this might potentially affect positivity by increased case finding. However, the effect of this difference in our before-after analysis was minimized by selection of participants with a high previous testing frequency (3.2 tests per year before PrEP vs 4.45 tests during PrEP), as well as by basing positivity calculations on a maximum of 1 test and 1 positive result per quarterly window. These data also demonstrate the very high STI testing frequency that can be achieved when MSM are taking PrEP, which may have public health benefits. Over longer periods of follow-up, in populations with low prior STI testing rates, STI incidence in men taking PrEP has been predicted to decline as increased PrEP-related testing substantially increases the detection and treatment of asymptomatic infection.^26^ Further modeling is needed using real-world PrEP and STI data to explore this question further because testing quarterly also has resource implications for health services and leads to increase antibiotic treatment, which is a concern as antimicrobial resistance is increasing.^27^

### Strengths and Limitations

A strength of this analysis is the large cohort of participants with prospective follow-up for up to 2 years enabling long-term trends in STIs to be examined. Most other reported studies have had a shorter follow-up.^28^ The other key strength is its use of long-term retrospective STI data using the ACCESS cohort, which has allowed a comparison of STI trends before and after PrEP use.

There are also potential limitations. First, infectious syphilis results were available from only a subset of participating public funded clinics with both clinical and laboratory data available. However, PrEP was free to all participants, irrespective of clinic type, which is likely to have reduced any selection bias. Second, the study population represents a high-risk group of men, and these findings may not be generalizable to all men starting PrEP. However, considering that contemporary international guidelines recommend PrEP to people at high risk of HIV infection, including in Europe and the US, and to people at risk of HIV infection in Australia, then our cohort represents an important target population of programs.^16,29,30^ Third, there is the potential for missed participant testing where tests for STIs were conducted outside of the ACCESS network. However, participant attendance at EPIC-NSW clinics for quarterly HIV and STI testing was a requirement of ongoing enrollment and continued prescription of PrEP.

## Conclusions

In New South Wales, Australia, STI incidence rates were already high and increased after men started PrEP, but there was no increase in STI test positivity after PrEP. Our findings demonstrate the importance of considering preexisting trends in STIs and testing when describing how PrEP use may affect STI incidence trends. The high STI positivity (52% of participants diagnosed with at least 1 STI in 12 months) among men taking PrEP highlights the importance of further efforts to control STIs among MSM.

## Notes

Supplement. **eTable 1.** Participant Testing Metrics by Stage of Follow-up **eTable 2.** Before-After Average Positivity for Participants With No Record of Prior PrEP Use and ≥2 Tests in Year Prior to PrEP **eTable 3.** Before-After Location Shift After Adjustment for Trend for Participants With No Record of Prior PrEP Use and ≥2 Tests in Year Prior to PrEP **eTable 4.** Before-After Trend in Positivity for Participants With No Record of Prior PrEP Use and ≥2 Tests in Year Prior to PrEP Excluding Adjustment for Location Shift **eTable 5.** Before-After Trend in Positivity and Location Shift for Participants With No Record of Prior PrEP Use and ≥2 Tests in Year Prior to PrEP Excluding Enrolment Window **eTable 6.** Before-After Trend in Positivity and Location Shift for Participants With No Record of Prior PrEP Use and ≥2 Tests in Year Prior to PrEP and Adjusted for Time Period of Enrolment
