# Supplementary material for: Comparison of Trends in Rates of Sexually Transmitted Infections Before vs After Initiation of HIV Preexposure Prophylaxis Among Men Who Have Sex With Men
Source: JAMA Netw Open. 2020 Dec 23;3(12):e2030806. doi: 10.1001/jamanetworkopen.2020.30806 (PMC7758809; doi:10.1001/jamanetworkopen.2020.30806)
Supplement: Supplement. — eTable 1. Participant Testing Metrics by Stage of Follow-up eTable 2. Before-After Average Positivity for Participants With No Record of Prior PrEP Use and ≥2 Tests in Year Prior to PrEP eTable 3. Before-After Location Shift After Adjustment for Trend for Participants With No Record of Prior PrEP Use and ≥2 Tests in Year Prior to PrEP eTable 4. Before-After Trend in Positivity for Participants With No Record of Prior PrEP Use and ≥2 Tests in Year Prior to PrEP Excluding Adjustment for Location Shift eTable 5. Before-After Trend in Positivity and Location Shift for Participants With No Record of Prior PrEP Use and ≥2 Tests in Year Prior to PrEP Excluding Enrolment Window eTable 6. Before-After Trend in Positivity and Location Shift for Participants With No Record of Prior PrEP Use and ≥2 Tests in Year Prior to PrEP and Adjusted for Time Period of Enrolment [file jamanetwopen-e2030806-s001.pdf]

Supplemental Online Content

McManus H, Grulich AE, Amin J, et al. Comparison of trends in rates of sexually transmitted infections before vs after initiation of HIV preexposure prophylaxis among men who have sex with men. *JAMA Netw Open*. 2020;3(12):e2030806.
doi:10.1001/jamanetworkopen.2020.30806

eTable 1. Participant Testing Metrics by Stage of Follow-up

eTable 2. Before-After Average Positivity for Participants With No Record of Prior PrEP Use and ≥2 Tests in Year Prior to PrEP

eTable 3. Before-After Location Shift After Adjustment for Trend for Participants With No Record of Prior PrEP Use and ≥2 Tests in Year Prior to PrEP

eTable 4. Before-After Trend in Positivity for Participants With No Record of Prior PrEP Use and ≥2 Tests in Year Prior to PrEP Excluding Adjustment for Location Shift

eTable 5. Before-After Trend in Positivity and Location Shift for Participants With No Record of Prior PrEP Use and ≥2 Tests in Year Prior to PrEP Excluding Enrolment Window

eTable 6. Before-After Trend in Positivity and Location Shift for Participants With No Record of Prior PrEP Use and ≥2 Tests in Year Prior to PrEP and Adjusted for Time Period of Enrolment

This supplemental material has been provided by the authors to give readers additional information about their work.

© 2020 McManus H et al. *JAMA Network Open.*

eTable 1. Participant Testing Metrics by Stage of Follow-up

Metric	Before-after cohort[a] (prior to PrEP)	Before-after cohort[a] (1year follow-up on PrEP)	p[b]	Before-after cohort[a] (2 years follow-up on PrEP)	p[b]
Number of positive results[c], N (%)					
0	1195 (50)	1172 (49)	0.51	895 (37)	<0.001
1	817 (34)	766 (32)	0.12	687 (29)	<0.001
2	310 (13)	339 (14)	0.22	411 (17)	<0.001
3 or more	82 (3)	127 (5)	0.00	411 (17)	<0.001
Duration between tests[d], mean (SD)	83.01 (64.11)	69.31 (42.37)	<0.001	73.82 (52.28)	<0.001
Number of tests per year[e], mean (SD)	3.22 (1.44)	4.45 (1.85)	<0.001	3.57 (1.79)	<0.001

PrEP=Pre-exposure prophylaxis

a. EPIC participants with testing after enrolment and no record of prior PrEP and with 2 or more test in the year prior to enrolment; 587/30,549 (1.9%) tests did not have specified anatomical site. Includes 20 participants who did not have a test in first year of follow-up on PrEP; 1,209/2,404 (50%) tested were positive ever in year prior to PreP, and 1,232/2,384 (52%) tested were positive ever in 1 year follow-up on PrEP.

b. T-test used for comparison of means with year prior to PrEP; chi-squared test used to compare count data with year prior to PrEP

c. limited to one positive test per quarter for any STI, denominator is number of individual participants with an STI test excluding baseline

d. Based on time between preceding and current test

e. Adjusted to annual rate based on complete quarters of follow-up prior to cohort censor date, excludes testing during window of enrolment

© 2020 McManus H et al. *JAMA Network Open.*

eTable 2. Before-After Average Positivity for Participants With no Record of Prior PrEP Use and ≥2 Tests in Year Prior to PrEP[a]

Disease	Site of infection	Positivity (95% CI)			
		Before PrEP	PrEP	RR (95% CI)	p[b]
Chlamydia	Anorectal	7.80 (7.18-8.42)	9.87 (9.32-10.43)	1.27 (1.15-1.40)	<0.001
	Pharyngeal	1.27 (1.02-1.53)	1.30 (1.10-1.50)	1.02 (0.79-1.31)	0.88
	Urethral	3.19 (2.80-3.58)	4.02 (3.67-4.37)	1.26 (1.09-1.47)	0.002
	Any site	10.32 (9.63-11.01)	13.62 (12.98-14.25)	1.32 (1.22-1.43)	<0.001
Gonorrhoea	Anorectal	4.28 (3.82-4.74)	5.54 (5.12-5.95)	1.29 (1.13-1.48)	<0.001
	Pharyngeal	6.00 (5.46-6.55)	5.57 (5.16-5.99)	0.93 (0.83-1.04)	0.22
	Urethral	1.88 (1.58-2.18)	2.45 (2.17-2.72)	1.30 (1.07-1.58)	0.008
	Any site	11.11 (10.39-11.82)	12.36 (11.75-12.97)	1.11 (1.03-1.21)	0.01
Chlamydia /Gonorrhoea	Anorectal	11.21 (10.47-11.96)	14.01 (13.34-14.67)	1.25 (1.15-1.36)	<0.001
	Pharyngeal	7.16 (6.56-7.76)	6.66 (6.20-7.12)	0.93 (0.84-1.04)	0.19
	Urethral	4.78 (4.31-5.26)	6.04 (5.61-6.47)	1.26 (1.12-1.43)	<0.001
	Any site	19.51 (18.56-20.46)	22.88 (22.06-23.71)	1.17 (1.10-1.25)	<0.001
Syphilis		1.85 (1.43-2.26)	1.61 (1.29-1.94)	0.88 (0.65-1.18)	0.39
Any	Any site	20.00 (19.04-20.95)	23.32 (22.49-24.15)	1.17 (1.10-1.24)	<0.001

PrEP=Pre-exposure prophylaxis; RR=rate ratio; CI=confidence interval

a. N=2,404. Positivity calculated using limit of 1 positive result and test per participant per quarter. Multivariate Poisson regression model adjusted for PrEP status, patent-level random effects.

b. Wald test

© 2020 McManus H et al. *JAMA Network Open.*

eTable 3. Before-After Location Shift After Adjustment for Trend for Participants With No Record of Prior PrEP Use and ≥2 Tests in Year Prior to PrEP[a]

**Disease**	**Site of infection**		
		**RR intercept (95%CI)**	**P[b]**
**Chlamydia**	Anorectal	1.09 (0.93-1.28)	0.30
	Pharyngeal	0.78 (0.51-1.20)	0.26
	Urethral	0.98 (0.77-1.26)	0.89
	Any site	1.05 (0.93-1.20)	0.42
**Gonorrhoea**	Anorectal	1.21 (0.98-1.49)	0.08
	Pharyngeal	1.05 (0.87-1.28)	0.61
	Urethral	1.35 (0.99-1.85)	0.06
	Any site	0.99 (0.87-1.12)	0.88
**Chlamydia/Gonorrhoea**	Anorectal	1.09 (0.96-1.24)	0.19
	Pharyngeal	1.00 (0.84-1.20)	0.98
	Urethral	1.11 (0.91-1.34)	0.30
	Any site	1.01 (0.92-1.10)	0.88
**Syphilis**		0.48 (0.28-0.82)	0.008
**Any**	Any site	1.00 (0.92-1.09)	0.96

PrEP=Pre-exposure prophylaxis; RR=rate ratio; CI=confidence interval

a. N=2,404. EPIC participants with testing after enrolment and no record of prior PrEP and with 2 or more test in the year prior to enrolment, 587/30,549 (1.9%) tests did not have specified anatomical site, including 263/12,733 (2.1%) before PrEP & 324/17,492 (1.8%) during PrEP , chi-squared test for difference p=0.121. Multivariable binomial-log regression adjusted for PrEP status and quarter of follow-up, patient-level random effects.

b. Wald test

© 2020 McManus H et al. *JAMA Network Open.*

eTable 4. Before-After Trend in Positivity for Participants With No Record of Prior PrEP Use and ≥2 Tests in Year Prior to PrEP Excluding Adjustment for Location Shift[a]

Disease	Site of infection	Before PrEP (change/quarter) (95% CI)	*P*[b]	After PrEP (change/quarter) (95% CI)	*P*[b]	RR trend[1] (95% CI)	*P*[b]
**Chlamydia**	Anorectal	1.11 (1.06-1.16)	<0.001	1.01 (0.99-1.03)	0.29	0.91 (0.86-0.97)	0.002
	Pharyngeal	1.02 (0.91-1.15)	0.74	1.02 (0.96-1.07)	0.57	1.00 (0.86-1.16)	0.96
	Urethral	1.13 (1.05-1.22)	0.001	1.01 (0.98-1.04)	0.53	0.89 (0.81-0.98)	0.02
	Any site	1.12 (1.08-1.16)	<0.001	1.02 (1.00-1.03)	0.017	0.91 (0.87-0.96)	<0.001
**Gonorrhoea**	Anorectal	1.10 (1.03-1.18)	0.003	1.01 (0.98-1.03)	0.61	0.91 (0.84-0.99)	0.03
	Pharyngeal	1.03 (0.98-1.09)	0.25	0.96 (0.94-0.99)	0.004	0.93 (0.87-1.00)	0.052
	Urethral	1.07 (0.98-1.17)	0.12	1.02 (0.98-1.06)	0.29	0.95 (0.85-1.06)	0.36
	Any site	1.07 (1.03-1.11)	<0.001	1.00 (0.99-1.02)	0.86	0.94 (0.89-0.98)	0.006
**Chlamydia/Gonorrhoea**	Anorectal	1.10 (1.06-1.14)	<0.001	1.01 (1.00-1.03)	0.19	0.92 (0.88-0.96)	<0.001
	Pharyngeal	1.03 (0.98-1.08)	0.28	0.97 (0.95-0.99)	0.009	0.94 (0.89-1.01)	0.07
	Urethral	1.11 (1.05-1.18)	<0.001	1.01 (0.99-1.03)	0.45	0.91 (0.85-0.98)	0.009
	Any site	1.08 (1.05-1.11)	<0.001	1.01 (1.00-1.02)	0.2	0.93 (0.90-0.96)	<0.001
**Syphilis**		0.99 (0.86-1.13)	0.84	1.03 (0.96-1.11)	0.36	1.05 (0.87-1.26)	0.62
**Any**	Any site	1.08 (1.05-1.11)	<0.001	1.01 (1.00-1.02)	0.21	0.93 (0.90-0.96)	<0.001

PrEP=Pre-exposure prophylaxis; RR=rate ratio; CI=confidence interval

a. N=2,404. EPIC participants with testing after enrolment and no record of prior PrEP and with 2 or more test in the year prior to enrolment, 587/30,549 (1.9%) tests did not have specified anatomical site, including 263/12,733 (2.1%) before PrEP & 324/17,492 (1.8%) during PrEP , chi-squared test for difference p=0.121. Multivariate binomial-log regression adjusted for quarter of follow-up, patient-level random effects.

b. Wald test

eTable 5. Before-After Trend in Positivity and Location Shift for Participants With No Record of Prior PrEP Use and ≥2 Tests in Year Prior to PrEP Excluding Enrolment Window[a]

Disease	Site of infection	before enrolment change/quarter-(95%CI)	P[b]	after enrolment change/quarter-(95%CI)	p[b]	RR trend (95%CI)	p[b]	RR intercept (95%CI)	p[b]
**Chlamydia**	Anorectal	1.06 (0.97-1.15)	0.19	1.00 (0.98-1.03)	0.85	0.95 (0.87-1.03)	0.22	1.19 (0.93-1.52)	0.17
	Pharyngeal	1.05 (0.86-1.27)	0.63	1.04 (0.97-1.12)	0.25	0.99 (0.81-1.22)	0.96	0.81 (0.45-1.49)	0.50
	Urethral	1.14 (1.00-1.30)	0.052	1.01 (0.97-1.05)	0.59	0.89 (0.77-1.02)	0.09	0.97 (0.67-1.39)	0.86
	Any site	1.09 (1.01-1.16)	0.02	1.01 (0.99-1.03)	0.17	0.93 (0.87-1.01)	0.07	1.12 (0.91-1.37)	0.28
**Gonorrhoea**	Anorectal	1.00 (0.89-1.12)	0.97	0.99 (0.96-1.02)	0.53	0.99 (0.88-1.12)	0.89	1.46 (1.04-2.05)	0.03
	Pharyngeal	0.97 (0.88-1.07)	0.52	0.96 (0.93-0.99)	0.009	0.99 (0.89-1.09)	0.80	1.23 (0.92-1.65)	0.16
	Urethral	0.97 (0.82-1.15)	0.71	0.99 (0.95-1.04)	0.67	1.02 (0.86-1.22)	0.81	1.54 (0.94-2.53)	0.08
	Any site	1.01 (0.95-1.08)	0.68	1.00 (0.98-1.02)	0.89	0.99 (0.92-1.06)	0.72	1.16 (0.96-1.41)	0.14
**Chlamydia /Gonorrhoea**	Anorectal	1.03 (0.96-1.11)	0.36	1.00 (0.98-1.02)	0.83	0.97 (0.90-1.04)	0.41	1.24 (1.02-1.52)	0.03
	Pharyngeal	0.98 (0.91-1.07)	0.71	0.97 (0.94-1.00)	0.04	0.98 (0.90-1.07)	0.71	1.13 (0.88-1.46)	0.34
	Urethral	1.07 (0.97-1.19)	0.18	1.00 (0.97-1.03)	0.92	0.93 (0.83-1.04)	0.20	1.16 (0.86-1.56)	0.35
	Any site	1.04 (0.99-1.09)	0.09	1.01 (0.99-1.02)	0.39	0.97 (0.92-1.01)	0.16	1.12 (0.98-1.29)	0.11
**Syphilis**		1.05 (0.82-1.35)	0.70	1.12 (1.02-1.23)	0.01	1.07 (0.82-1.39)	0.64	0.54 (0.25-1.17)	0.12
**Any**	Any site	1.04 (0.99-1.09)	0.10	1.01 (0.99-1.02)	0.33	0.97 (0.92-1.02)	0.19	1.12 (0.98-1.28)	0.10

PrEP=Pre-exposure prophylaxis; RR=rate ratio; CI=confidence interval

a. N=2,404. Positivity calculated using limit of 1 positive result and test per participant per quarter, Multivariable binomial-log regression adjusted for PrEP status and quarter of follow-up, patient-level random effects. Baseline quarter excluded from model fit.

b. Wald test

© 2020 McManus H et al. *JAMA Network Open.*

eTable 6. Before-After Trend in Positivity and Location Shift for Participants With No Record of Prior PrEP Use and ≥2 Tests in Year Prior to PrEP and Adjusted for Time Period of Enrolment[a]

Disease	Site of infection	trend before enrolment change per quarter (95%CI)	p	trend after enrolment change per quarter (95%CI)	p	Risk ratio of trend (95%CI)	p	Risk ratio of intercept (95%CI)	p
**Chlamydia**	Anorectal	1.09 (1.03-1.16)	0.001	1.00 (0.97-1.02)	0.84	0.91 (0.86-0.97)	0.003	1.10 (0.94-1.29)	0.25
	Pharyngeal	1.06 (0.93-1.22)	0.38	1.04 (0.98-1.12)	0.22	0.98 (0.85-1.14)	0.826	0.78 (0.50-1.20)	0.25
	Urethral	1.13 (1.04-1.24)	0.004	1.01 (0.97-1.05)	0.70	0.89 (0.81-0.98)	0.014	0.99 (0.78-1.26)	0.94
	Any site	1.11 (1.06-1.16)	<0.001	1.01 (0.99-1.03)	0.22	0.91 (0.87-0.96)	<0.001	1.06 (0.93-1.21)	0.391
**Gonorrhoea**	Anorectal	1.07 (0.99-1.15)	0.10	0.98 (0.94-1.01)	0.19	0.92 (0.84-0.10)	0.045	1.23 (1.99-1.53)	0.05
	Pharyngeal	1.02 (0.96-1.09)	0.49	0.95 (0.92-0.98)	0.002	0.93 (0.86-1.00)	0.037	1.07 (0.88-1.30)	0.48
	Urethral	1.02 (0.91-1.13)	0.79	1.00 (0.95-1.04)	0.86	0.98 (0.87-1.10)	0.744	1.34 (0.98-1.84)	0.07
	Any site	1.07 (1.02-1.12)	0.002	1.00 (0.98-1.02)	0.96	0.93 (0.89-0.98)	0.005	0.99 (0.88-1.13)	0.94
**Chlamydia/ Gonorrhoea**	Anorectal	1.08 (1.03-1.13)	0.001	1.00 (0.98-1.02)	0.71	0.92 (0.88-0.97)	0.001	1.10 (0.97-1.25)	0.14
	Pharyngeal	1.03 (0.97-1.08)	0.39	0.96 (0.93-0.99)	0.01	0.94 (0.88-1.00)	0.048	1.02 (0.85-1.21)	0.85
	Urethral	1.09 (1.02-1.17)	0.01	1.00 (0.97-1.03)	0.95	0.92 (0.85-0.99)	0.019	1.11 (0.91-1.34)	0.30
	Any site	1.08 (1.05-1.12)	<0.001	1.01 (0.99-1.02)	0.461	0.93 (0.90-0.96)	<0.001	1.01 (0.93-1.10)	0.82
**Syphilis**		1.10 (0.94-1.29)	0.25	1.11 (1.02-1.22)	0.02	1.01 (0.84-1.21)	0.905	0.48 (0.28-0.84)	0.009
**Any**	Any site	1.08 (1.05-1.11)	<0.001	1.01 (0.99-1.02)	0.40	0.93 (0.90-0.96)	<0.001	1.00 (0.92-1.09)	0.98

PrEP=Pre-exposure prophylaxis; RR=rate ratio; CI=confidence interval

a. N=2,404. EPIC participants with testing after enrolment and no record of prior PrEP and with 2 or more test in the year prior to enrolment, 587/30,549 (1.9%) tests did not have specified anatomical site, including 263/12,733 (2.1%) before PrEP & 324/17,492 (1.8%) during PrEP , chi-squared test for difference p=0.121. Multivariable binomial-log regression adjusted for PrEP status, enrolment period [May-16 to June-16 (early), July-16 to December-16 (middle), January-17 to April-18 (late)] and quarter of follow-up, patient-level random effects.

b. Wald test

© 2020 McManus H et al. *JAMA Network Open.*
